# Clinical data classification with noisy intermediate scale quantum computers

**DOI:** 10.1038/s41598-022-05971-9

**Published:** 2022-02-03

**Authors:** S. Moradi, C. Brandner, C. Spielvogel, D. Krajnc, S. Hillmich, R. Wille, W. Drexler, L. Papp

**Affiliations:** 1 Center for Medical Physics and Biomedical Engineering, Medical University of Vienna, Währinger Gürtel 18-20, 1090 Vienna, Austria; 2 Division of Nuclear Medicine, Medical University of Vienna, Vienna, Austria; 3 Institute for Integrated Circuits, Johannes Kepler University Linz, Linz, Austria; 4 Software Competence Center Hagenberg GmbH, Hagenberg, Austria

**Keywords:** Computer science, Quantum physics, Medical research

## Abstract

Quantum machine learning has experienced significant progress in both software and hardware development in the recent years and has emerged as an applicable area of near-term quantum computers. In this work, we investigate the feasibility of utilizing quantum machine learning (QML) on real clinical datasets. We propose two QML algorithms for data classification on IBM quantum hardware: a quantum distance classifier (qDS) and a simplified quantum-kernel support vector machine (sqKSVM). We utilize these different methods using the linear time quantum data encoding technique (${\mathrm{log}}_{2}N$) for embedding classical data into quantum states and estimating the inner product on the 15-qubit IBMQ Melbourne quantum computer. We match the predictive performance of our QML approaches with prior QML methods and with their classical counterpart algorithms for three open-access clinical datasets. Our results imply that the qDS in small sample and feature count datasets outperforms kernel-based methods. In contrast, quantum kernel approaches outperform qDS in high sample and feature count datasets. We demonstrate that the ${\mathrm{log}}_{2}N$ encoding increases predictive performance with up to + 2% area under the receiver operator characteristics curve across all quantum machine learning approaches, thus, making it ideal for machine learning tasks executed in Noisy Intermediate Scale Quantum computers.

## Introduction

Quantum technologies promise to revolutionize the future of information and computation using quantum devices to process massive amounts of data. To date, considerable progress has been made from both software and hardware points of view. Many researches are underway to simplify quantum algorithms^1–8^ in order to implement them on existing, so-called Noisy Intermediate Scale Quantum (NISQ) computers^9^. As a result, small quantum devices based on photons, superconductors, or trapped ions are capable of efficiently running scalable quantum algorithms^6,7,10^. Quantum Machine Learning (QML) is a particularly interesting approach, as it is suited for existing NISQ architectures^11–15^. While conventional machine learning is generally applied to process large amounts of data, many research fields cannot provide such large datasets. One example is medical research, where collecting cohorts that represent certain characteristics of diseases routinely results in small datasets^16^. NISQ devices can efficiently execute algorithms with shallow depth and a low number of qubits^9^. Therefore, it appears logical to exploit the potential of QML executed on NISQ devices incorporating clinical datasets.

However, the execution of QML algorithms in the form of practical quantum gate operations is non-trivial. First, the classical data needs to be encoded into quantum states. For this purpose, prior QML algorithms assume that a quantum random access memory (QRAM) device for storing the data is present^17^. Nevertheless, to date, such practical devices are not available. Second, since the output of quantum algorithms are obviously quantum states, the efficient classical bits of information must be extracted through quantum measurements. To date, various classical data encoding approaches have been proposed^6,7,18–21^. In particular, encoding classical numerical features into quantum states has the advantage to utilize ${\mathrm{log}}_{2}N$ number of qubits (a.k.a. linear time encoding) in relation to $N$ number of input features^18–21^. This approach allows to utilize NISQ devices with a small number of qubits and to minimize quantum noise, while at the same time maintaining quantum speedup^14^. In contrast, to date, this approach in combination with quantum machine learning appears to be underrepresented.

In light of the above proceedings, we hypothesize that clinically-relevant quantum prediction models can be built on NISQ devices employing the ${\mathrm{log}}_{2}N$ encoding, having prediction performances comparable to classic ML approaches.

In our work, we propose two quantum machine learning approaches that rely on the ${\mathrm{log}}_{2}N$ encoding approach, thus, not requiring the presence of a fault-tolerant quantum circuit for implementation of quantum RAM^17^. Previously proposed techniques for estimation of the inner product with Hadamard Test and Swap Test assume that there is a quantum RAM or a quantum circuit that store both index of data and their values^22,23^. To construct a quantum database (QDB) from classical data, $\left(n+m+1\right)$-qubits are required for $M$ sample and $N$-feature counts, where $n={\mathrm{log}}_{2}N$, $m={\mathrm{log}}_{2}M$, and 1 is considered as qubit register^24^. In contrast, the ${\mathrm{log}}_{2}N$ encoding technique utilizes only $n$ qubit and $O\left(Mn\right)$ steps to classically access to data without allocating extra qubits to the index of entries of dataset. First, we demonstrate a simple and efficient quantum distance classifier (qDC) executable on existing NISQ devices. Second, we present a simplified quantum-kernel SVM (sqKSVM) approach using quantum kernels which can be executed once without optimization instead of twice with optimization as in case of the quantum-kernel SVM (qKSVM) approach^6,7^.

In order to test our hypothesis, we demonstrate the performance of the qDC and the sqKSVM approaches using real clinical data and compare their performances to qKSVM, as well as to classic computing counterparts such as k-nearest neighbors^25^ and classic support vector machines^26^.

## Results

### Dataset

This study incorporated three open-access clinical datasets that have been presented and evaluated in various contexts^27–29^. Each dataset underwent redundancy reduction by correlation matrix analysis^30^ followed by a tenfold cross-validation split with a training-validation ratio of 80–20%^16^. Training sets of the folds were subjects of feature ranking analysis^31^ and the highest-ranking eight as well as 16 (if available) features were selected for further analysis. The resulted dataset configurations were analyzed by class imbalance ratios and the quantum advantage score (a.k.a. difference geometry)^20^ for quantum kernel methods. Table 1 demonstrates the characteristics of the data configurations as well as the results of the imbalance ratio and the quantum advantage scores (for estimation of the quantum advantage scores ${(g}_{CQ})$, see Appendix E of the supplementary material).

**Table 1:** Clinical datasets utilized for the study with their sample and selected feature count as well as their imbalance ratios and quantum advantage scores ${(g}_{CQ})$.

Dataset	#Samples	Imbalance Ratio	#Features	${g}_{CQ}$	Reference
Pediatric Bone Marrow Transplant 2-year survival	134	0.33	8	0.40	^27^
			16	0.60	
Wisconsin Breast Cancer Malign-vs-benign	569	0.37	8	1.30	^28^
			16	3.50	
Heart Failure Mortality	300	0.5	8	0.42	^29^

Given a two-class dataset, the imbalance ratio ($IR$) is $IR=x/y$, where $x$ is the number of minority class and $y$ is the total number of samples. Furthermore, ${g}_{CQ}$ measures the similarities of quantum kernel and linear classical kernel functions of the same dataset.

### Encoding strategies

This study relies on the data encoding strategy which uses sequences of Pauli-Y gate rotations (${R}_{y}$) and $CNOT$ gates (see Appendix A of the supplementary material) to result in a number of ${\mathrm{log}}_{2}N$ encoding qubits^18,19,21^. ${R}_{y}$ puts each qubit $q$ in a superposition state ${R}_{y}\left(2\theta \right)\left|q\rangle \right.=\mathrm{cos}\left(\theta \right)\left|0\rangle \pm \mathrm{sin}\left(\theta \right)\right.\left|1\rangle \right.$ and $CNOT$ s entangle qubits. The data encoding feature map with the application of ${R}_{y}$ and $CNOT$ is given by^32^

1 $$\varphi :\overrightarrow{x}\to \left|\varphi \left(\overrightarrow{\theta }\right)\rangle \right.\langle \left.\varphi \left(\overrightarrow{\theta }\right)\right|$$

where $\left|\varphi \left(\overrightarrow{\theta }\right)\rangle \right.={U}_{\varphi \left(\overrightarrow{\theta }\right)}{\left|0\rangle \right.}^{\otimes {\mathrm{log}}_{2}N}$. In Eq. (1), $\varphi \left(\overrightarrow{\theta }\right)$ is the encoding map from the Euclidean space to the Hilbert space and ${U}_{\varphi \left(\overrightarrow{\theta }\right)}$ is the model circuit for data encoding, which maps ${\left|0\rangle \right.}^{\otimes {\mathrm{log}}_{2}N}$ to another ket vector of input data $\left|\varphi \left(\overrightarrow{\theta }\right)\rangle \right.$. To find a relationship between the input data and $\theta$, see Appendix A of the supplementary material.

In contrast, previously proposed quantum ML-specific encoding utilizes a block of the Hadamard gates followed by a block of Pauli-Z gate rotations (${R}_{z}$) are applied to each qubit^7^. To entangle the qubits, nearest neighbor $CNOT$ s are also applied. The features of data samples are considered as angles of ${R}_{z}$ rotations and the required number of qubits for data encoding are equal to the number of features. The data encoding feature map with the application of the Hadamard, ${R}_{z}$ and $CNOT$ is given by^7^

2 $$\varphi :\overrightarrow{x}\to \left|\varphi \left(\overrightarrow{x}\right)\rangle \right.\langle \left.\varphi \left(\overrightarrow{x}\right)\right|$$

where $\left|\varphi \left(\overrightarrow{x}\right)\rangle \right.={U}_{\varphi \left(\overrightarrow{x}\right)}{H}^{\otimes N}{\left|0\rangle \right.}^{\otimes N}$. ${U}_{\varphi \left(\overrightarrow{x}\right)}$ is the model circuit for N features data encoding.

In order to compare the predictive performance of the above two data encoding strategies, the qDC, the sqKSVM and the qKSVM (see Appendix C of the supplementary material) approaches were compared utilizing a number of $N=8$ features. This analysis was executed using the Pennylane simulator environment^33^, while the sqKSVM was also evaluated on the IBMQ Melbourne machine (see “Methods”). Table 2 demonstrates the cross-validation area under the receiver operator characteristics curve (AUC) performance values of the quantum ML algorithms in relation to the ${\mathrm{log}}_{2}N$ and $N$ encoding qubit strategies.

**Table 2:** Comparison of the cross-validation AUC performance for different data encodings.

Dataset	qDC	qKSVM	sqKSVM	sqKSVM*	qubits
Pediatric Bone Marrow Transplant 2YS	0.62	0.63	0.62	0.61	${\mathrm{log}}_{2}N$
	0.61	0.63	0.61	0.59	$N$
Wisconsin Breast Cancer Malign-vs-benign	0.92	0.92	0.88	0.87	${\mathrm{log}}_{2}N$
	0.90	0.91	0.87	0.85	$N$
Heart failure Mortality	0.62	0.51	0.51	0.50	${\mathrm{log}}_{2}N$
	0.60	0.51	0.51	0.50	$N$

The qDC, qKSVM, and sqKSVM run on Pennylane simulator for $N=8$. For the ${\mathrm{log}}_{2}N$ encoding, $N$ features are encoded into ${\mathrm{log}}_{2}N$ qubits with sequences of Pauli-Y gate rotations (${R}_{y}$) and $CNOT$ s. In another strategy, $N$ features are encoded into $N$ qubits with sequences of the Hadamard gates, Pauli-Z gate (${R}_{z}$) rotations followed by nearest neighbor $CNOT$ s.

*The sqKSVM was also executed on the IBMQ Melbourne machine for reference comparison.

### Quantum and classic machine learning predictive performance evaluation

The quantum distance classifier (qDC) first calculates the distance between the state vector of a test sample and each state vector of the training sample in set $P$ and set $Q$ and, then, assigns a label of the test sample to the label of the closest set. In the qDC, we divide the training set, with $M$ number of samples, based on their labels $\left\{a, b\in R\right\}$ into two subset $\left\{P\right\}$ and $\left\{Q\right\}$, where $\left\{P\right\}$ contains only label $a$ with the number of samples $MP$ and $\left\{Q\right\}$ contains only label $b$ with the number of samples $MQ$ with $MP+MQ=M$. The task is to determine the label of the given test sample $\left\{{y}_{k}\right\}$, if ${y}_{k}=a$ or ${y}_{k}=b$. Mathematically, if $\left|v\rangle \right.$ is the state vector of the test sample as well as $\left|u\rangle \right.\in P$ and $\left|w\rangle \right.\in Q$, then the label of $\left|v\rangle \right.$ is determined by ${y}_{k}=a$, if $min\left(\left|\left|u\rangle \right.-\left|v\rangle \right.\right|\right) \le min\left(\left|\left|w\rangle \right.-\left|v\rangle \right.\right|\right)$, otherwise ${y}_{k}=b$. The distance between the vectors is given by^8^ i.e.

3 $$\left|\left|u\rangle \right.-\left|v\rangle \right.\right|= \left|\left|u\rangle \right.\right|\left|\left|v\rangle \right.\right|-\langle u|v\rangle$$

where $\left|\bullet \right|$ is the norm ${l}_{2}$ of a vector. Therefore, the task is to calculate the inner product $\langle u|v\rangle$ with a quantum computer.

The two different approaches to estimate $\langle u|v\rangle$ with quantum computers are Hadamard Test^22^ and the Swap Test^23^. For the simplified quantum kernel SVM (sqKSVM), we first need to note that the standard form of the quantum kernelized binary classifiers is

4 $$\tilde{y }=sgn\left(\sum_{i=1}^{M}{{{y}_{i}\alpha }_{i}}^{*}K\left({\overrightarrow{x}}_{i},\overrightarrow{\tilde{x }}\right)\right)$$

where $\tilde{y }$ is the unknown label, ${y}_{i}$ is the label of the $i$ ^th^ training sample, ${{\alpha }_{i}}^{*}$ is the $i$ ^th^ component of the support vector ${\overrightarrow{\alpha }}^{*}=\left({{\alpha }_{1}}^{*}, {{\alpha }_{2}}^{*},\dots , {{\alpha }_{M}}^{*}\right)$, $M$ is the number of training data, and $K\left({\overrightarrow{x}}_{i},\overrightarrow{\tilde{x }}\right)$ is the kernel matrix of all the training-test pairs.

For a given dataset $D= {\left\{\left({\overrightarrow{x}}_{i}, {y}_{i}\right):{\overrightarrow{x}}_{i}\in {R}^{M}, {y}_{i}\in \left\{-1, 1\right\} \right\}}_{i=1,\dots ,M}$, one option to bypass the drawbacks of the qKSVM algorithm (see Appendix C of the supplementary material) as presented in^6,7^ is to set uniform weight ${{\alpha }_{i}}^{*}=1$, in case of $IR=0.5$ (balanced dataset). Otherwise, ${{\alpha }_{i}}^{*}=IR$ for the majority class and ${{\alpha }_{j}}^{*}=1-IR$ for the minority class. Thresholding the value $\sum_{i=1}^{M}{{{y}_{i}\alpha }_{i}}^{*}K\left({\overrightarrow{x}}_{i},\overrightarrow{\tilde{x }}\right)$ yields the binary output as following

5 $$\tilde{y }=\left\{\begin{array}{cc} 1& \sum_{i=1}^{M}{{{y}_{i}\alpha }_{i}}^{*}K\left({\overrightarrow{x}}_{i},\overrightarrow{\tilde{x }} \right)\ge 0\\ -1& else\end{array}\right.$$

In Eq. (5), $K\left({\overrightarrow{x}}_{i},\overrightarrow{\tilde{x }}\right)$ is defined as (see Appendix F of the supplementary material)

6 $$K\left({\overrightarrow{x}}_{i},\overrightarrow{\tilde{x }} \right)={\left|\langle u|v\rangle \right|}^{2}$$

The dataset configurations were utilized to estimate the performance of quantum and classic machine learning algorithms incorporated in this study. Performance estimation was done by confusion matrix analytics^34^. Prediction models were built based on the given training subset, followed by evaluating the respective validation subsets of each fold. Average area under the receiver operator characteristics curve (AUC) was calculated across validation cases for each predictive model. To build predictive models, quantum ML approaches included the qDC, the sqKSVM and the qKSVM (see Appendix C of supplementary material) were utilized. Classic machine learning approaches were k-nearest neighbors (ckNN)^25^ and support vector machines (cSVM)^26^. See Table 3 for the comparison of cross-validation AUC performances of quantum and classic computing algorithm within the dataset configurations.

**Table 3:** Comparison of the cross-validation AUC performance with QML and ML algorithms.

Dataset	#Features	sqKSVM	qKSVM	qDC	cSVM	ckNN
Pediatric Bone Marrow Transplant 2YS	8	0.61	0.63	0.60	0.64	0.61
	16	0.66	0.69	0.64	0.71	0.64
Wisconsin Breast Cancer Malign-vs-benign	8	0.87	0.92	0.91	0.89	0.90
	16	0.88	0.93	0.90	0.89	0.93
Heart Failure Mortality*	8	0.50	0.51	0.60	0.53	0.58

For all QML algorithms, $N$ features are encoded into ${\mathrm{log}}_{2}N$ qubits with sequences of Pauli-Y gate rotations (${R}_{y}$) and $CNOT$ s. All QML algorithms were executed on the IBMQ Melbourne machine.

*Heart failure has no 16-feature variant, since the maximum number of features are 13.

### Estimation of the probability of errors rate

**Figure 1:**
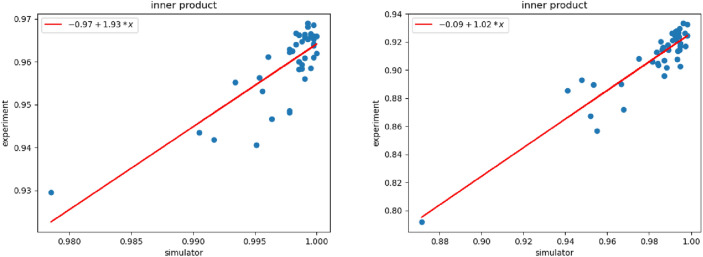
Scatter diagrams of simulator inner products vs. experiment inner products for both the train state vectors and test state vectors. This data corresponds to the Wisconsin Breast Cancer dataset with 8 (left) and 16 (right) features. The red lines represent optimal fit lines based on least-squared regression.

Our experimental demonstrations are performed on the 15-qubit IBMQ Melbourne processor based on superconducting transmon qubits. The experiment has been conducted on the Wisconsin Breast Cancer dataset with 8 and 16 features, given, that this dataset provided the highest predictive cross-validation performance. On the NISQ device and simulator, each circuit is run with a fixed number of measurement shots (= 8192). We plot scatter diagrams for the inner product values from the simulator and the inner product from the NISQ device in Fig. 1. To show the correlation between the experimental and the simulator values of the inner products, we also fit optimal lines using least square regression in Fig. 1. To measure the difference between the inner products from the simulator and the inner product from the NISQ device, the root mean square error (RMSE) was calculated. The value of RMSE was 0.039 (3.9%) and 0.075 (7.5%) for 8 and 16 feature counts, respectively (Fig. 1). Therefore, the fidelities of the quantum circuits on the quantum cloud device were estimated 96% and 92.5% for the 8 and 16 feature counts, respectively. For more details of the experiment see Appendix G of the Supplementary material.

The depolarizing noise model represents a linear relationship between the ideal (simulator) and the noisy (experiment) values of the inner products based on Eq. (14) in “Methods”. Nevertheless, the slope of the fit lines in Fig. 1 show that the depolarizing noise model cannot estimate the true value of probability of error rate ($\lambda$). This is due to gate errors^35^ that are originated from miscalibration of quantum Hardware, not being covered by the depolarizing noise model.

## Discussion

In this study, we aimed to investigate the effect of two encoding strategies in various quantum machine learning-built clinical prediction models. Next to prior quantum machine learning approaches, we also proposed two methods specifically designed for the ${\mathrm{log}}_{2}N$ encoding approach.

Our results demonstrate that the ${\mathrm{log}}_{2}N$ encoding in combination with low-complexity quantum machine learning approaches provides comparable or better results than the $N$ encoding approach with previously-proposed quantum machine learning methods. This advantage was demonstrated not only in a simulator environment, but also utilizing NISQ devices. The low algorithmic quantum complexity also aims towards building prediction models that may be easier to interpret in the future, especially in light of the high complexity of classic machine learning approaches^36^. In contrast, it is important to emphasize, that the proposed quantum machine learning processes are also applicable in big data, given, that calculating the inner product of quantum states in NISQ devices can be done efficiently with the ${\mathrm{log}}_{2}N$ encoding approach^21,22^. The ${\mathrm{log}}_{2}N$ data encoding is also more robust against noise compared to the $N$ data encoding, since it uses less number of noisy qubits of the NISQ device to estimate the inner product of quantum states^10^.

After encoding data from classical Euclidean space into quantum Hilbert space, the distance between data points may increase or decrease, which has implications in case of kernel methods^20^. The ${g}_{CQ}$ score can represent, whether distances between data points would increase or decrease after data encoding. For further explanations see Supplemental Appendix E.

When feature count increases, ${g}_{CQ}$ increases as well, because quantum state vectors of input features become closer due to the high dimensionality property of the Hilbert space. Higher feature count significantly influences performance in a positive way if ${g}_{CQ}$ is < 1 (e.g. + 5–6% AUC in the Pediatric bone marrow dataset). It has been shown that classical ML models are competitive or outperform quantum ML approaches when ${g}_{CQ}$ is small^20^. Nevertheless, we demonstrated that when ${g}_{CQ}>1$, higher feature count does not contribute much to the performance increase (e.g. 1% difference in the Wisconsin breast cancer dataset). It is important to point out that a high ${g}_{CQ}$ (> 1) alone does not mean that the dataset is not ideal for kernel-based quantum machine learning. Specifically, the highest AUC of 0.93 was achieved in the 16 feature counts Wisconsin breast cancer dataset, while it also demonstrated the highest ${g}_{CQ}$, which also confirms prior findings^20^. In contrast, the same dataset in the classic SVM resulted in 0.89 AUC. We hypothesize that this phenomenon is due to the high sample count of the Wisconsin breast cancer dataset (M = 569). In general, the imbalance ratio of the datasets did not appear to be correlated with predictive performance. The ${\mathrm{log}}_{2}N$ increased AUC with up to 2% compared to the $N$ encoding when comparing the execution of the quantum machine learning approaches using simulation environment. This behavior was also identifiable with executions in NISQ devices, in case of kernel methods and the qDC. We hypothesize that lower AUC performance for the $N$ encoding method in the simulator environment and NISQ device is due to higher number of qubits which likely lead to lower value of inner products. This is in line with the findings in^20^.

In general, the qKSVM demonstrated 2–5% higher AUC compared to the sqKSVM. The relative performance increase of the qKSVM was in relation to sample count and feature count. Specifically, the qKSVM showed an average 2% higher AUC with small sample count (Heart failure and Pediatric bone datasets), while it had 5% higher AUC in the Wisconsin breast cancer dataset. Nevertheless, both the qKSVM and the sqKSVM increased its AUC with double feature counts in the small Pediatric bone marrow dataset. This level of performance increase was not identifiable in the larger Wisconsin breast cancer dataset. Classic SVM demonstrated similar properties in relation to higher feature counts in small datasets^20^, while it was outperformed by the qKSVM in the large Wisconsin breast cancer dataset.

In conclusion, quantum SVM approaches benefit from higher feature count in general, where the qKSVM—due to relying on optimization—has a particular benefit compared to the sqKSVM. In contrast, the sqKSVM algorithm reduces the time complexity of the qKSVM algorithm significantly, which may be advantageous in case of large datasets on NISQ devices. In the large Wisconsin breast cancer dataset, the qDC demonstrated higher performance compared to the sqKSVM, especially in small feature counts (0.91 AUC vs 0.87 AUC in the qDC and the sqKSVM respectively in 8 features). The qDC resulted in the highest AUC of 0.60 across all other quantum (0.50–0.51 AUC) and classic machine learning (0.53–0.58 AUC) approaches in the Heart failure dataset. We hypothesize that this is due to the distribution characteristics of the samples belonging to the two subclasses in the feature space, which challenges classification with kernel methods. Generally, the performance of the executed quantum and classic machine learning approaches are comparable within the collected cohorts (Table 3).

According to our findings, quantum distance approaches can provide high performance with small feature and sample counts, which is particularly ideal for NISQ devices. In contrast, quantum kernel methods appear to provide high performance with high feature and sample counts. We demonstrated that the ${\mathrm{log}}_{2}N$ encoding strategy allows to execute quantum ML algorithms for highly dimensional clinical datasets on low qubit count NISQ devices. In general, quantum machine learning benefits from utilizing the ${\mathrm{log}}_{2}N$ encoding strategy, as it increases predictive performance and reduces execution time in NISQ devices, while keeping model complexity lower. Our experiments also pointed out an important implication of how noise shall be estimated. As such, the depolarizing noise model cannot cover gate errors.

We consider our findings of high importance in relation to building future quantum ML prediction models in NISQ devices for clinically-relevant cohorts and beyond.

## Methods

All experiments of this study were performed in accordance with the respective guidelines and regulations of the open-access data sources this study relied on. For details, see section “Access”.

### ***Estimation of the inner product ***$\langle {\varvec{u}}|{\varvec{v}}\rangle$*** and***${\left|\langle {\varvec{u}}|{\varvec{v}}\rangle \right|}^{2}$

**Figure 2:**
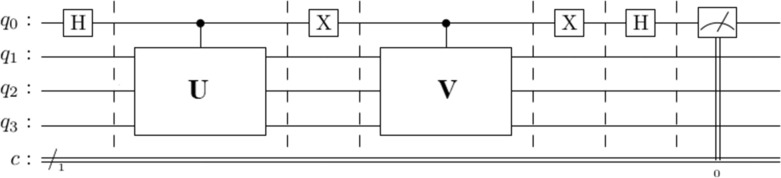
Quantum circuit computes the real part of the inner product $\langle u|v\rangle$. The Hadamard gate puts the ancilla qubit (${q}_{0}$) into uniform superposition. A single-controlled unitary gate entangles the exited state of the ancilla qubit with the training data state vector ($\left|u\rangle =U\left|{q}_{1}{q}_{2}{q}_{3}\rangle \right.\right.$). The $X$ gate flips the ancilla qubit. Another single unitary controlled gate entangles the state vector of the test data ($\left|v\rangle =V\left|{q}_{1}{q}_{2}{q}_{3}\rangle \right.\right.$) with the excited state of the ancilla qubit. A second $X$ gate flips the ancilla qubit. The Hadamard gate on the ancilla qubit interferences train and test data state vectors. The ancilla qubit is measured using a Pauli-$Z$ gate. The real value of $\langle u|v\rangle$ is estimated from Eq. (9). The measurement gate is done by a Pauli-$Z$ gate and $$Z=\left(\begin{array}{cc}1& 0\\ 0& -1\end{array}\right)$$.

Figure 2 shows the quantum circuit for estimation of the real part of $\langle u|v\rangle$ with the Hadamard Test.

To estimate the real part of $\langle u|v\rangle$ on the quantum computer with the Hadamard Test, the training and test data needs to be prepared in a quantum state as

7 $$\frac{1}{\sqrt{2}}\left({\left|0\rangle \right.}_{a}\left|v\rangle \right.+{\left|1\rangle \right.}_{a}\left|u\rangle \right.\right)$$

where $\left|u\rangle \right.$ and $\left|v\rangle \right.$ are the quantum states for the train and test datasets, respectively.

Then the Hadamard gate on the ancilla qubit interferences the training vector $\left|u\rangle \right.$ with the test vector $\left|v\rangle \right.$

8 $$\frac{1}{2}\left({\left|0\rangle \right.}_{a}(\left|v\rangle +\left|u\rangle \right.)\right.+{\left|1\rangle \right.}_{a}(\left|v\rangle -\right.\left|u\rangle )\right.\right)$$

Finally, the measuring quantum state given in Eq. (8) in the computational basis ${\left|0\rangle \right.}_{a}$ gives probability as

9 $$\mathrm{Pr}(\left|{0\rangle }_{a})\right.=\frac{\left(1+Re\left(\langle u|v\rangle \right)\right)}{2}$$

where $\mathrm{Pr}$ is the value of the probability of measurement on the ${\left|0\rangle \right.}_{a}$ state of Eq. (8) and $\langle u|u\rangle =\langle v|v\rangle =1$. Since our datasets are real values $Re\left(\langle u|v\rangle \right)=\langle u|v\rangle$. See Appendix H of the Supplementary material for details of the estimation of the inner product on the IBMQ Melbourne machine with the Hadamard Test.

**Figure 3:**
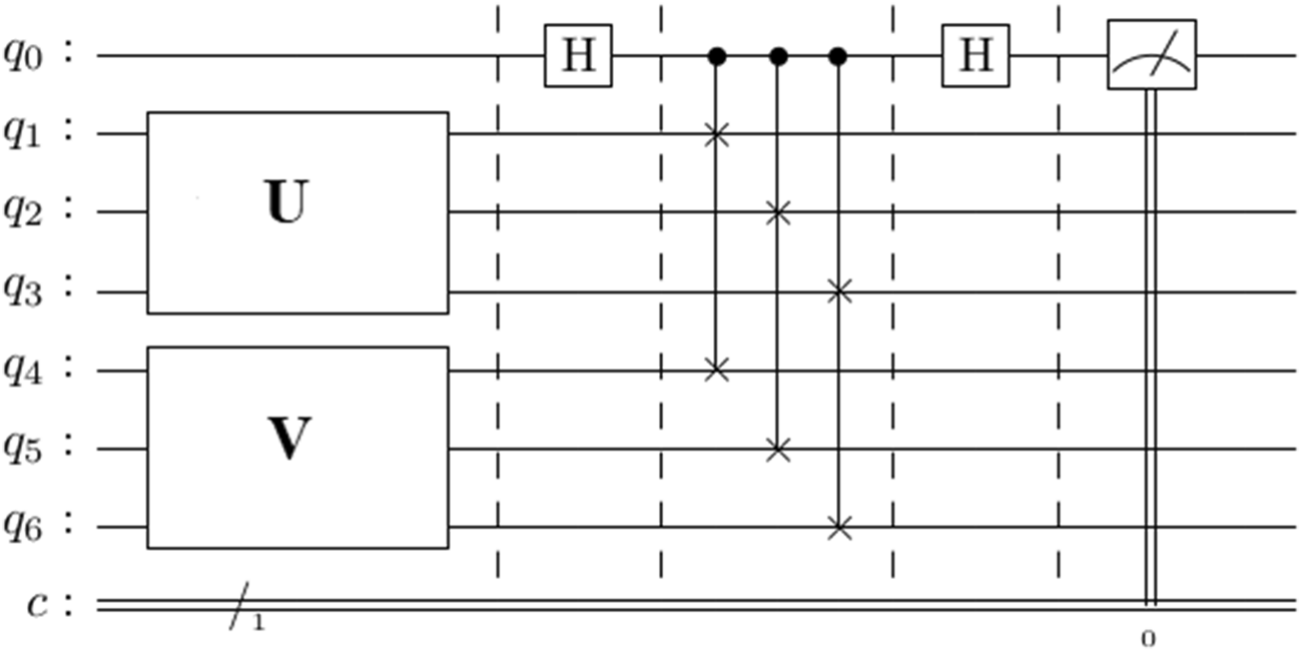
Quantum Circuit to compute ${\left|\langle u|v\rangle \right|}^{2}$. The model circuits encode train and test data into quantum states $\left|u\rangle =U\left|{q}_{1}{q}_{2}{q}_{3}\rangle \right.\right.$ and $\left|v\rangle =V\left|{q}_{4}{q}_{5}{q}_{6}\rangle \right.\right.$. The Hadamard gate on the ancilla qubit (${q}_{0}$) generates a superposition of the quantum state including the train and test datasets. The application of the single-controlled swap gates with the ancilla qubit as the control results in an entangled state of Eq. (10). Another Hadamard gate on the ancilla qubit interferences $\left|u\rangle \left|v\rangle \right.\right.$ and $\left|v\rangle \left|u\rangle \right.\right.$. The ancilla qubit on the $\left|0\rangle \right.$ state is measured in the Z basis. Therefore, the value of ${\left|\langle u|v\rangle \right|}^{2}$ can be obtained from Eq. (12).

The inner product $\langle u|v\rangle$ can also be estimated on a quantum computer with the Swap Test (see Fig. 3). The Hadamard gate is applied on the ancilla qubit to create a superposition of $\left|u\rangle \left|v\rangle \right.\right.$, i.e.

10 $$\frac{1}{\sqrt{2}}\left({\left|0\rangle \right.}_{a}\left|u\rangle \left|v\rangle +{\left|1\rangle \right.}_{a}\left|u\rangle \left|v\rangle\right) \right.\right.\right.\right.$$

The application of the single-controlled swap gates on the state given in Eq. (10) entangles the ancilla qubit with $\left|u\rangle \left|v\rangle \right.\right.$. The resulted entangled quantum state is $\frac{1}{\sqrt{2}}\left({\left|0\rangle \right.}_{a}\left|u\rangle \left|v\rangle +{\left|1\rangle \right.}_{a}\left|v\rangle \left|u\rangle \right.\right.\right.\right.\right)$. Then, another Hadamard gate interferences the product state of the state vectors of the training and the test i.e.

11 $$\frac{1}{2}\left({\left|0\rangle \right.}_{a}\left(\left|u\rangle \left|v\rangle \right.\right.+\left|v\rangle \left|u\rangle \right.\right.\right)+{\left|1\rangle \right.}_{a}\left(\left|u\rangle \left|v\rangle \right.\right.-\left|v\rangle \left|u\rangle \right.\right.\right)\right)$$

Measuring the quantum state given in Eq. (11) in the computational basis yields ${\left|0\rangle \right.}_{a}$ with the probability

12 $$\mathrm{Pr}(\left|{0\rangle }_{a})\right.= \frac{\left(1+{\left|\langle u|v\rangle \right|}^{2}\right)}{2}$$

where $\mathrm{Pr}$ is the value of the probability of measurement on the ${\left|0\rangle \right.}_{a}$ state of Eq. (11). See Appendix D of the Supplementary material for details of the estimation of the inner product on the IBMQ Melbourne machine with the Hadamard Test.

### Simplified quantum kernel support vector machine

**Figure 4:**
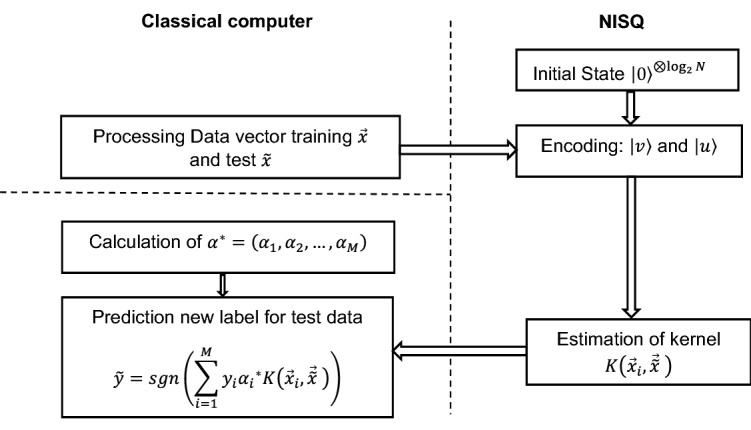
Schematic of the sqKSVM for data classification algorithm. First, the training data vector $\overrightarrow{x}$ and test $\tilde{x }$ are prepared on a classical computer. Next, the original training data and test data are encoded into quantum states followed by computing the kernel matrix of all pairs of the training-test data $K\left({\overrightarrow{x}}_{i},\overrightarrow{\tilde{x }}\right)$ with a NISQ computer. If ${\alpha }^{*}= \left({{\alpha }_{1}}^{*}, {{\alpha }_{2}}^{*}, \dots ,{{\alpha }_{M}}^{*}\right)$ are considered to be a solution of the support vector, the binary classifier can be constructed based on Eq. (5).

The quantum Support Vector Machine algorithm is proposed in^37^ for big data classification. They show exponential speedup for their algorithm via quantum mechanically access to data. Nevertheless, this approach is not ideal for NISQ devices^9^. To date, two separate qKSVM approaches are proposed for data classification via classical access to data^6,7^. In these approaches, the quantum circuits must run twice on the quantum computer and a cost function needs to be optimized on the classical computer to compute the support vector^7^. We propose a simplified version qKSVM called sqKSVM as shown in Fig. 4.

### Software and hardware

For classical machine learning algorithms, we use classical machine learning (CML) libraries of scikit-learn^38^. Pennylane-Qiskit^33^ is used for quantum circuit simulation and quantum experiment for designing quantum computing programs. Pennylane-Qiskit 0.13.0 plugin integrates the Qiskit quantum computing framework to the Pennylane simulator.

**Figure 5:**
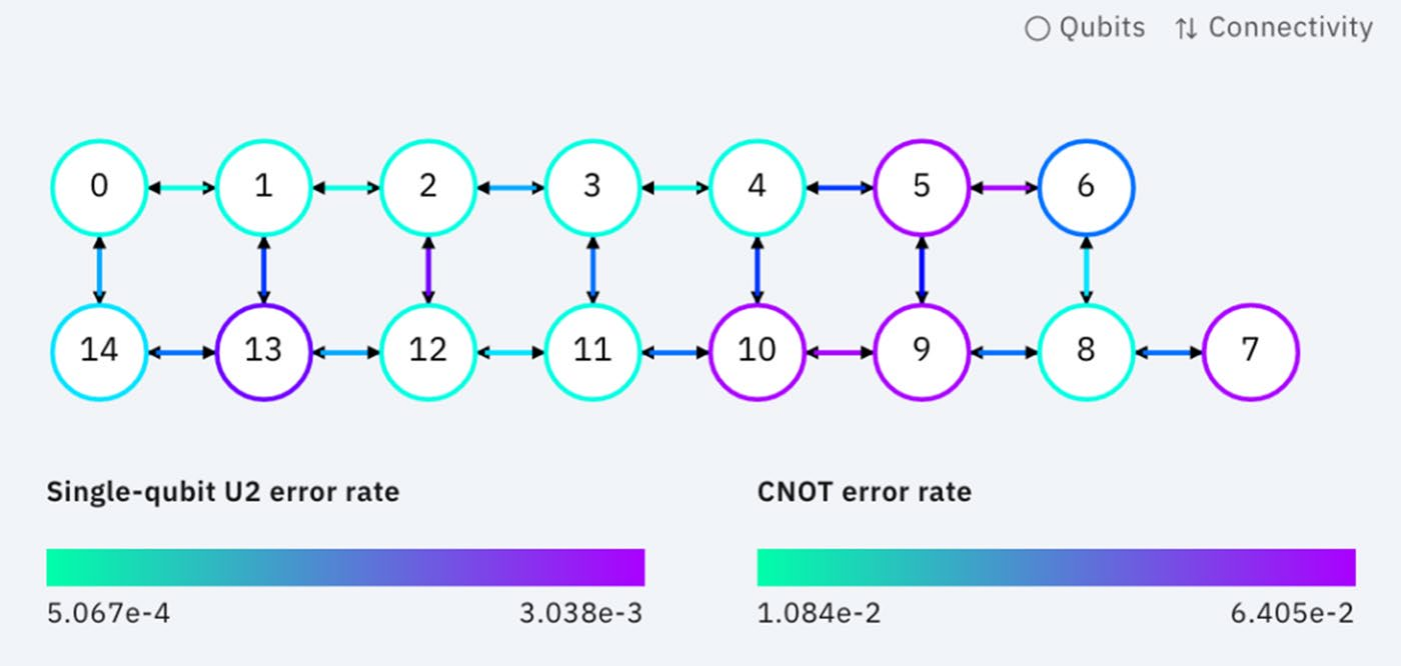
Topology and coupling map of the IBMQ Melbourne (https://quantum-computing.ibm.com/services). Single-qubit error rate is the error induced by applying the single-qubit gates. $CNOT$ error is the error of the only two-qubit $CNOT$ gates. Each circle represents a physical superconducting qubit and each $\leftrightarrow$ shows coupling between neighbor qubits.

For executing quantum algorithms on existing quantum computers, this study relied on IBM’s remote quantum machines (https://quantum-computing.ibm.com/) that can run quantum programs with noisy qubits. Since IBM quantum computers only support single-qubit gate and two-qubit $CNOT$ gate operations, complex gate operations must be decomposed into elementary supported gates before mapping the quantum circuit on noisy hardware. Owing to the specific architecture of IBM quantum computers, all two-qubit $CNOT$ gate operations must satisfy the constraints imposed by the coupling map^39^, i.e., if ${q}_{i}$ is the control qubit and ${q}_{j}$ is the target qubit, $CNOT({q}_{i}, {q}_{j})$ can only be applied if there is coupling between ${q}_{i}$ and ${q}_{j}$. In case of running the QML algorithms on the quantum computer, we choose the 15-qubits IBMQ Melbourne machine with the supported gates $I$, ${U}_{3}$, and $CNOT$, where $I$ is identity single-qubit gate, ${U}_{3}$ is single-qubit arbitrary rotation gates with $CNOT$ as two-qubit gate. Figure 5 shows the coupling map of the IBMQ Melbourne with its gate error rates.

### Depolarizing noise model

A simple model to describe incoherent noise is the depolarizing noise model. For a $n$ qubit pure quantum state $\left|u\rangle \right.$, the depolarizing noise operator (channel) leads to a loss of information with probability $\lambda$ and with probability $\left(1-\lambda \right)$ the system is left untouched^40^. The state of the system after this noise is

13 $${\epsilon }_{\lambda }\left(\rho \right)= \left(1-\lambda \right)\rho +\lambda \frac{I}{{2}^{n}}$$

where ${\epsilon }_{\lambda }$ denotes the noise channel, $\rho =\left|u\rangle \right.\langle \left.u\right|$ is a density matrix, $\lambda$ is the probability of error rate that depends on NISQ devices, the gate operations, and the depth of the quantum circuit, and $I$ is the (${2}^{n}\times {2}^{n}$) identity matrix.

The expectation value of observable $O$ for a state represented by a density matrix $\rho$ is given by

14 $$\overline{\langle O\rangle }=tr\left[\epsilon \left(\rho \right)O\right]=\left(1-\lambda \right)\langle O\rangle +\frac{\lambda }{{2}^{n}}tr\left(O\right)$$

where $\overline{\langle O\rangle }$ is the noisy expectation value and $\langle O\rangle$ is the noiseless expectation value^41^.

## Supplementary Information

Supplementary Information.

## Data Availability

The source code of the implemented quantum algorithms can be accessed by the link: https://github.com/sassan72/Quantum-Machine-learning. For classical machine learning executions, this study relied on the scikit-learn library^38^: https://scikit-learn.org/stable/. All included open-access datasets are accessible through the following links: Pediatric bone marrow transplant dataset^27^: https://archive.ics.uci.edu/ml/datasets/Bone+marrow+transplant%3A+children. Wisconsin breast cancer dataset^28^: https://archive.ics.uci.edu/ml/datasets/Breast+Cancer+Wisconsin+(Diagnostic). Heart failure dataset^29^: https://www.kaggle.com/andrewmvd/heart-failure-clinical-data.
