# Supplementary material for: Clinical data classification with noisy intermediate scale quantum computers
Source: Sci Rep. 2022 Feb 3;12:1851. doi: 10.1038/s41598-022-05971-9 (PMC8814029; doi:10.1038/s41598-022-05971-9)
Supplement: Supplementary file 1 — Supplementary Information. [file 41598_2022_5971_MOESM1_ESM.pdf]

Clinical Data Classification With
Noisy Intermediate Scale Quantum Computers
Supplemental

S. Moradi[1], C. Brandner[1], C. Spielvogel[2], D. Krajnc[1], S. Hillmich[3], R. Wille[3,4], W. Drexler[1], L. Papp[1]*

[1] Center for Medical Physics and Biomedical Engineering, Medical University of Vienna, Vienna, Austria

[2] Division of Nuclear Medicine, Medical University of Vienna, Vienna, Austria

[3]Institute for Integrated Circuits, Johannes Kepler University Linz, Linz, Austria

[4]Software Competence Center Hagenberg GmbH, Hagenberg, Austria

Appendix A: Data encoding

The idea behind data encoding is to use sequences of the multi-controlled rotations. Given a classical normalized data vector $\vec{a} = (a_1, a_2, a_3, a_4, a_5, a_6, a_7, a_8)$, the problem is how to build the quantum circuit to encode the 8-dimensional vector $\vec{a}$ into a state vector of three qubit register,

$$\sum_{i=0}^{7} a_{i+1}|i\rangle, \tag{A1}$$

where, in our case, a_{i+1} are real numbers. In [1], the reverse problem is considered which is about mapping $|\psi\rangle$ to the ground state $|0 \dots 0\rangle$ by using multi-controlled rotations. Since quantum gate operations are unitary and reversible, we can use the routine in [1] for data encoding by inverting each gate operation [2]. Fig. S1 shows the quantum circuit for mapping $|\psi\rangle$ of equation (A1) to $|000\rangle$. In order to implement the quantum circuit of Fig. S1 on NISQ efficiently, we need to decompose controlled R_y-rotations into single R_y-rotations and $CNOT$ gates as shown in Fig. S2. To implement a uniformly multi controlled rotation using single qubit rotations and $CNOT$ gates, a decomposition based on Gray codes is used [1,3]. For this purpose, the multi-controlled rotation

angles β in Fig. S1 have to be converted into a set of non-controlled rotation angles θ in Fig. S2 (see equation (A3)). To this end, first, equation (A2) computes the rotation angles required to implement the uniformly controlled R_y-rotations applied to the qubits in Fig. S1.

$$\beta_j^k = 2\sin^{-1}\left[\frac{\sqrt{\sum_{l=0}^{2^{k-1}}\left|a_{(2j-1)2^{k-1}+l}\right|^2}}{\sqrt{\sum_{l=0}^{2^k}\left|a_{(j-1)2^k+l}\right|^2}}\right], \tag{A2}$$

where $j = 1, 2, \ldots, 2^{n-k}$, $k = 1, 2\ 3$, and $n(= 3)$ is the number of qubits[1]. The angles θ are obtained from β:

$$\begin{pmatrix} \theta_1^k \\ \theta_2^k \\ \vdots \\ \theta_{2^k-1}^k \\ \theta_{2^k}^k \end{pmatrix} = M^{(k)} \begin{pmatrix} \beta_1^k \\ \beta_2^k \\ \vdots \\ \beta_{2^k-1}^k \\ \beta_{2^k}^k \end{pmatrix}, \tag{A3}$$

where $M_{ij}^{(k)} = 2^{-k}(-1)^{g_{i-1}.b_{j-1}}$, b_j is the binary representation of the decimal j, and g_j is the grey code. Moreover, the dot product $g_{i-1}.b_{j-1}$ is the bitwise inner product, for example, $(01).(11) = 0 \times 1 + 1 \times 1 = 1$. Table S1 shows the values b_j the binary decimal, and the values of g_i the grey code.

i	g_i
0	000
1	001
2	011
3	010
4	110
5	111
6	101
7	100

j	b_j
0	000
1	001
2	010
3	011
4	100
5	101
6	110
7	111

Table S1. The value of grey code (left) and the value of the binary decimal (right) for three-bit version.

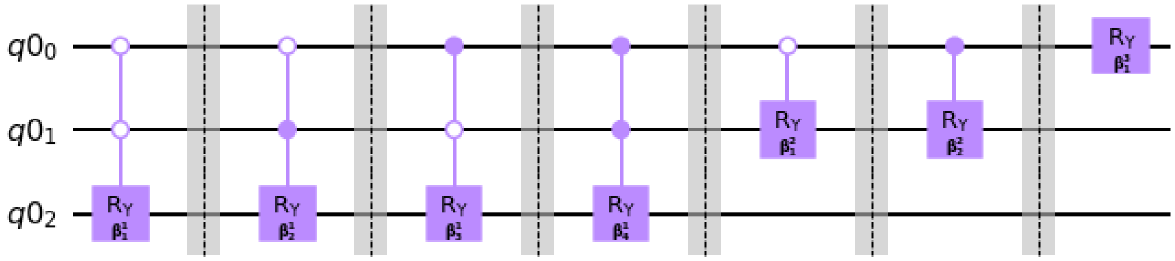

Figure S1. Gate operations for mapping $|\boldsymbol{\psi}\rangle$ to the ground state $|\mathbf{000}\rangle$. The white circle indicates a control on qubit being in state $|0\rangle$, and the full circle a control on qubit being in state $|1\rangle$. β_j^ks are angles for R_y-rotation gates, which can be obtained from equation (A2).

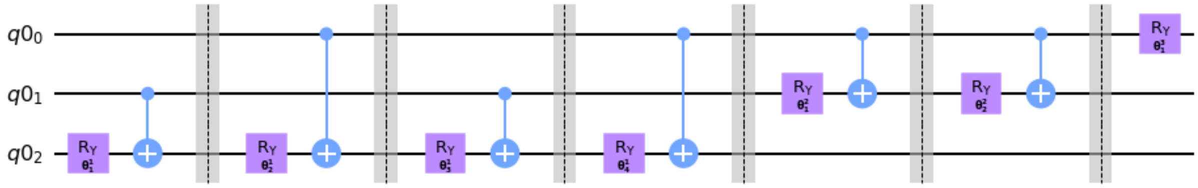

Figure S2. Quantum circuit for mapping $|\boldsymbol{\psi}\rangle$ to the ground state $|\mathbf{000}\rangle$ after gate decomposition. θ_j^ks are angles for R_y-rotation gates. Equation (A3) shows the relationship between β_j^k and θ_j^k angles.

Now the reverse can generate the two-qubit state $|\psi\rangle$ (equation (A1)) from the ground state $|000\rangle$ as shown in Fig. S3.

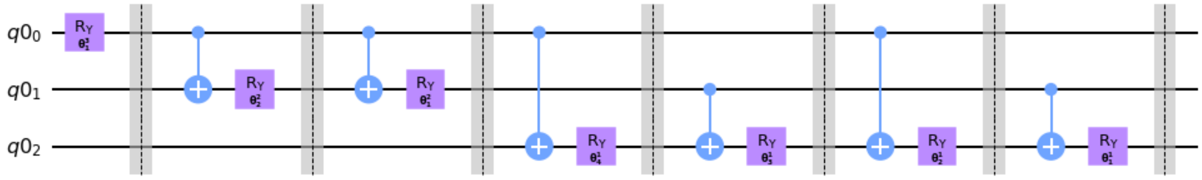

Figure S3. Quantum circuit for mapping $|\mathbf{000}\rangle$ to $|\boldsymbol{\psi}\rangle$. This quantum circuit can encode a vector with 8-components into a quantum state with three qubits.

three-controlled rotations are also required, as shown in Fig. S4.

Equation (A2) computes the rotation angles required to implement the uniformly controlled R_y-rotations applied to the qubits in Fig. S4 for $k = 1, 2, 3, 4$, and $n(= 4)$ is the number of qubits[1].

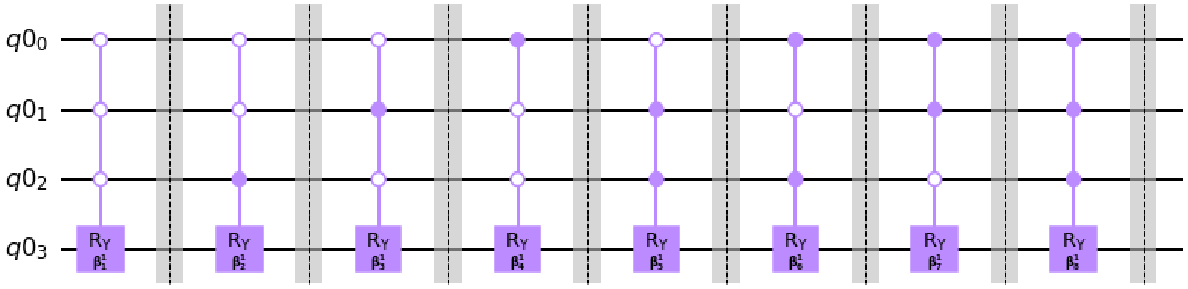

Figure S4. Quantum circuit of the uniformly three-controlled rotations about y axis. The white circles indicate a control on qubit being in state $|0\rangle$, and the full circles a control on qubit being in state $|1\rangle$.

Fig. S5 shows the efficient gate decompositions for the uniformly controlled rotations in Fig. S4 based on Grey code [1].The angles θ are obtained from β with equation (A3). Table S2 shows the values b_j the binary decimal j, and the grey values g_i.

i	g_i
0	0000
1	0001
2	0011
3	0010
4	0110
5	0111
6	0101
7	0100
8	1100
9	1101
10	1111
11	1110
12	1010
13	1011
14	1001
15	1000

j	b_j
0	0000
1	0001
2	0010
3	0011
4	0100
5	0101
6	0110
7	0111
8	1000
9	1001
10	1010
11	1011
12	1100
13	1101
14	1110
15	1111

Table S2. The value of grey code (left) and the value of the binary decimal (right) for four-bit version.

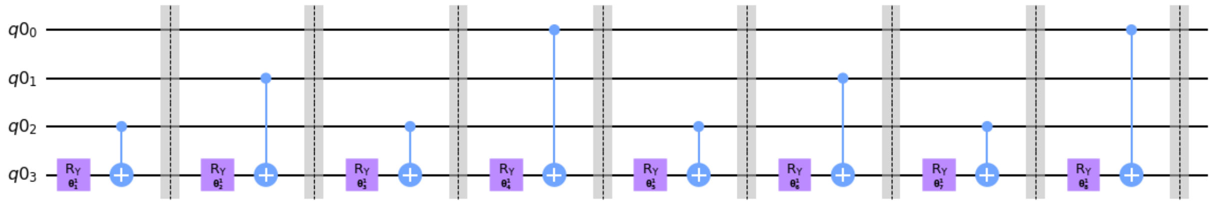

Figure S5. Gate decomposition for uniformly three-controlled rotations of Fig. S4. The quantum circuit only includes single R_y- rotations and $CNOT$ gates.

Appendix B: Gate decompositions

Compilation of the Hadamard Test and the Swap Test are computationally expensive on NISQ devices due to existence of Toffoli gate, single control R_y rotations, and three-qubits controlled swap gates. Fig. S6(b) shows the efficient decomposition of Toffoli gate (Fig. S6(a)) into Hadamard (H), T, $T^\dagger$, and $CNOT$ gates. In Fig. S7(a), one single control R_y-rotation gate is decomposed into 8 single R_Z and R_X rotations and 2 $CNOT$s.

(a) **(b)**

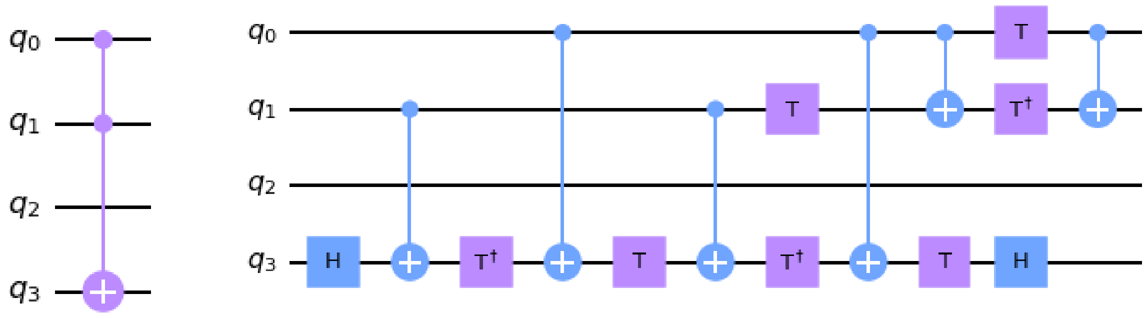

Figure S6. (a) Shows Toffoli gate $CCNOT(q_0, q_1, q_3)$. (b) A decomposition of $CCNOT(q_0, q_1, q_3)$.

(a) **(b)**

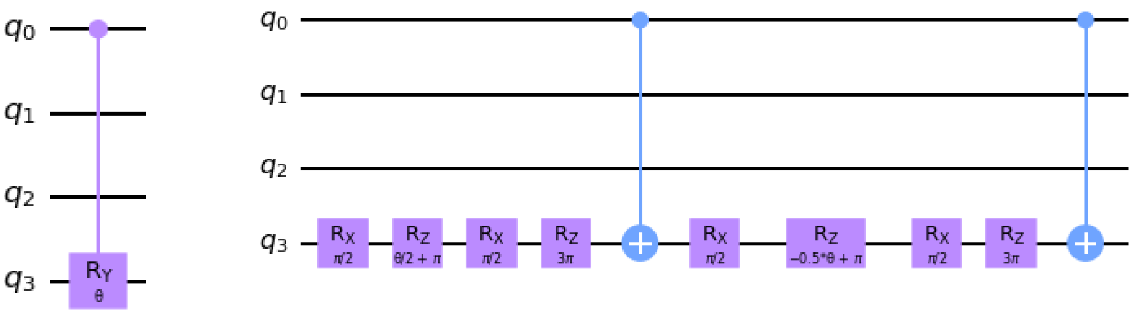

Figure S7. (a) shows single control R_y-rotation gate. (b) A decomposition of single control R_y-rotation gate.

For datasets with 16 features, there are some $CNOT$ which cannot be implemented directly on IBMQ Melbourne machine due to coupling constraints. For example, $CNOT(q_0, q_4)$ must be decomposed before execution the quantum circuit on quantum computer as shown in Fig. S8(b).

(a) **(b)**

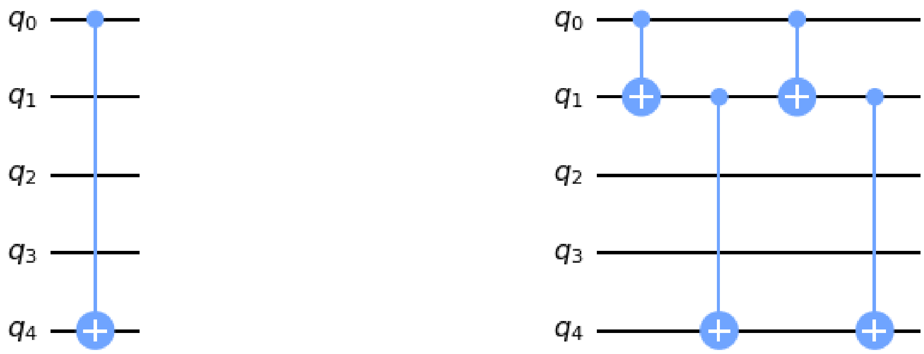

Figure S8. (a) Shows $CNOT(q_0, q_4)$ generated from decomposition Toffoli gate for dataset with 16 features. (b) A decomposition of $CNOT(q_0, q_4)$ into executable $CNOT$s.

The only computationally expensive gate operations for the SWAP Test are three-qubits controlled swap gates (See Fig. S9(a)). One single $CSWAP(q_0, q_3, q_6)$ is decomposed into 11 single-qubit gates and 7 $CNOT$s which is shown in Fig. S9(b). Some $CNOT$s, which have been highlighted with the dash box in Fig. S9(b), don't fulfil the coupling map constraints of IBMQ Melbourne machine. They must be decomposed into executable $CNOT$s as shown in Fig. S8(b).

(a) **(b)**

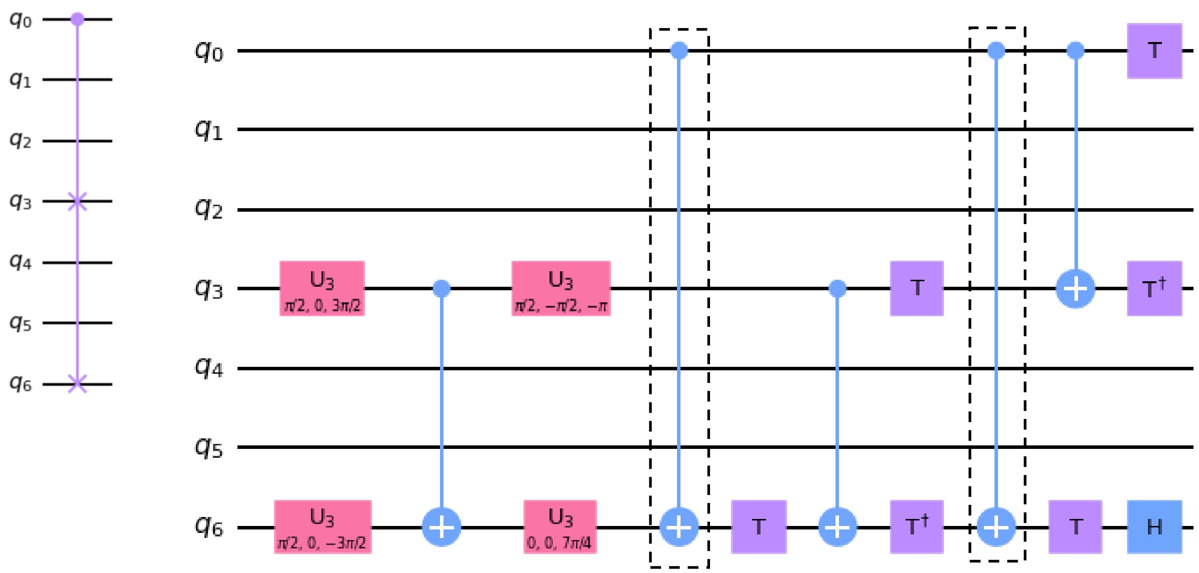

Figure S9. (a) shows control swap gate $CSWAP(q_0, q_3, q_6)$ for dataset with 8 features. (b) A decomposition of $CSWAP(q_0, q_3, q_6)$.

Appendix C: quantum Kernel Support Vector Machine (qKSVM) algorithm

Fig. S10 shows the schematic of qKSVM algorithm [4,5]. In this protocol, the NISQ device is used twice. Given M training data samples $D = \{(\vec{x}_i, y_i): \vec{x}_i \in R^M, y_i \in \{-1, 1\}\}_{i=1,\ldots,M}$, the task is to find label $\tilde{y}$ of a given test data vector $\tilde{\boldsymbol{x}}$. First the original training data vector $\vec{x}$ and their labels are prepared on the classical computer. Next, the original training data are encoded into quantum states using quantum gate operations followed by computing kernel matrix of all pair of the training data ($K(\vec{x}_i, \vec{x}_j)$). This step runs on the NISQ computer. Then the computed quantum kernel matrix $K(\vec{x}_i, \vec{x}_j)$ is fed into the classical computer to find the support vector based on the quantum kernel matrix of the training data and the training labels by maximizing [5],

$$L_D(\alpha) = \sum_{i=1}^{M} \alpha_i - \frac{1}{2} \sum_{i,j=1}^{M} y_i y_j \alpha_i \alpha_j K(\vec{x}_i, \vec{x}_j), \quad \text{(C1)}$$

subjected to these two constraints $\sum_{i=1}^{N} \alpha_i y_i = 0$ and $\alpha_i \geq 0$ for each i. In equation (C1), y_i, y_j, $K(\vec{x}_i, \vec{x}_j)$, and M are the label of ith training sample, the label j^{th}training sample, the kernel matrix

of training data, and the number of training samples, respectively. If the support vector $\alpha^* = (\alpha_1{}^*, \alpha_2{}^*, \ldots, \alpha_M{}^*)$ is considered to be a solution of equation (C1), the binary classifier can be constructed,

$$\tilde{y} = sgn\left(\sum_{i=1}^{M} y_i \alpha_i{}^* K(\vec{x}_i, \vec{\tilde{x}}) + b\right) \tag{C2}$$

where $K(\vec{x}_i, \tilde{\boldsymbol{x}})$ is the quantum kernel matrix of the training-test pairs and b can be obtained by solving $\sum_j y_j \alpha_j{}^* K(\vec{x}_i, \vec{x}_j) + b = y_i$. Equation (C2) predicts the label $\tilde{y}$ for the given test data. Quantum computer is used for the second time to evaluate $K(\vec{x}_i, \tilde{\boldsymbol{x}})$.

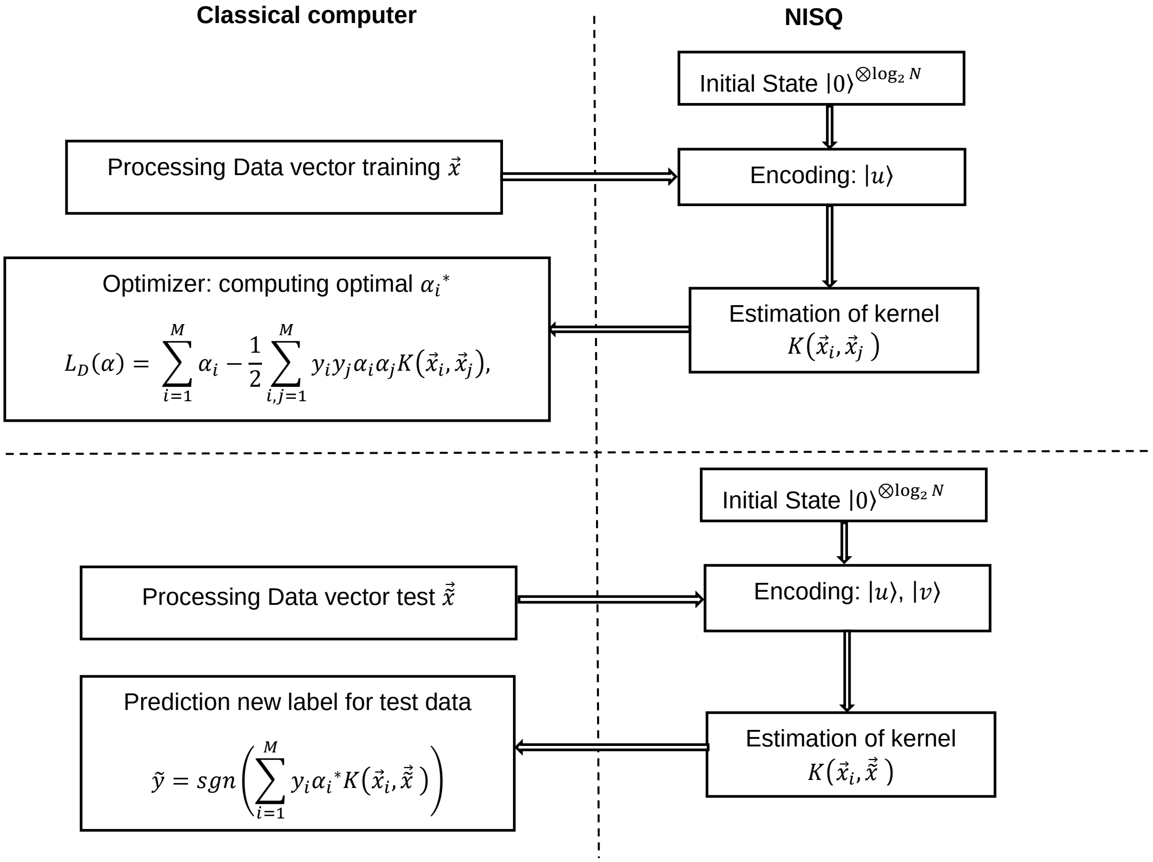

Figure S10. Schematic of qKSVM algorithm for data classification. NISQ device must run twice to estimate $K(\vec{x}_i, \vec{x}_j)$ and $K(\vec{x}_i, \vec{\tilde{x}})$.

Appendix D: Implementation of the Swap Test on IBMQ Melbourne machine

In order to map the circuit for the Swap Test on IBMQ Melbourne machine, first, we design the circuit of the Swap Test in a simulator [6]. Then all single control swap gates must be

decomposed into the basis gates U_3, $CNOT$, $T^\dagger$, H, and T. $CNOT(q_i, q_j)$s with the distance $d = |q_i - q_j| > 3$ don't satisfy the coupling constraints of IBMQ Melbourne machine [7,8]. The efficient decomposition approach is to consider physical qubits path Q_2 (as root), Q_{12}, Q_{11}, Q_{10}, Q_9, Q_8, and Q_7 (goal node) from Fig. 4 of the manuscript as simulator qubits q_0, q_1, q_2, q_3, q_4, q_5, and q_6. The decomposition of $CNOT(q_0, q_6)$ is given by

$$CNOT(Q_2, Q_7) = CNOT(Q_2, Q_{12})CNOT(Q_{12}, Q_7)CNOT(Q_2, Q_{12})CNOT(Q_{12}, Q_7) \quad \text{(D1)}$$

With more decomposition for $CNOT(Q_{12}, Q_7)$ in equation (D1)

$$CNOT(Q_{12}, Q_7) = CNOT(Q_{12}, Q_{11})CNOT(Q_{11}, Q_7)CNOT(Q_{12}, Q_{11})CNOT(Q_{11}, Q_7), \quad \text{(D2)}$$

and from equation (D2) $CNOT(Q_{11}, Q_7)$ must be decomposed to fulfil the coupling constraints

$$CNOT(Q_{11}, Q_7) = CNOT(Q_{11}, Q_{10})CNOT(Q_{10}, Q_7)CNOT(Q_{11}, Q_{10})CNOT(Q_{10}, Q_7) \quad \text{(D3)}$$

Appendix E: Estimation of the quantum advantage score for kernel-based algorithms

In order to link data encoding and the quantum kernel function to the quantum prediction advantage, **the quantum advantage score (**the geometric difference) is used which only depends on the dataset, but is independent of the labels. The geometric difference measures the similarities of different kernel functions of the same dataset.

The geometric difference machine learning models based on classical kernel and quantum kernel is defined by [9]:

$$g_{CQ} = g(K^C \| K^Q) = \sqrt{\left\| \sqrt{K^Q}(K^C)^{-1}\sqrt{K^Q} \right\|_\infty}, \quad \text{(E1)}$$

where K^C, K^Q, and $\|.\|_\infty$are, the classical kernel matrix, the quantum kernel, and the spectral norm of the resulting matrix $\sqrt{K^Q}(K^C)^{-1}\sqrt{K^Q}$, respectively.

The physical meaning of g_{CQ} is explained as follows:

A kernel matrix is a semi definite matrix whose diagonal entries are 1 and off-diagonal entries are less than 1. The off-diagonal entries of a kernel matrix can be seen as measures of distance between data points in the same feature space. Mathematically, if $K(x_i, x_j)$, an off-diagonal entry of a kernel matrix, is large when the distance between x_i and x_j is small.

Fig. S11(a) and S11(b) show the positions of three data points A, B, and C in classical and quantum feature spaces, respectively, with the arrows representing the distances between data points.

After encoding data from classical Euclidean space into quantum Hilbert space, the distance between data points increases. From the kernel point of view, the entries of classical kernel matrix are larger compared to the entries of the quantum kernel matrix. For example, if the classical kernel matrix corresponds to Fig. S11(a) is

$$K^C = \begin{pmatrix} 1 & 0.9 & 0.8 \\ 0.9 & 1 & 0.7 \\ 0.8 & 0.7 & 1 \end{pmatrix}$$

and the quantum kernel matrix corresponds to Fig. S11(b) is

$$K^Q = \begin{pmatrix} 1 & 0.3 & 0.6 \\ 0.3 & 1 & 0.5 \\ 0.6 & 0.5 & 1 \end{pmatrix}$$

then the $g_{CQ} = 2.6$.

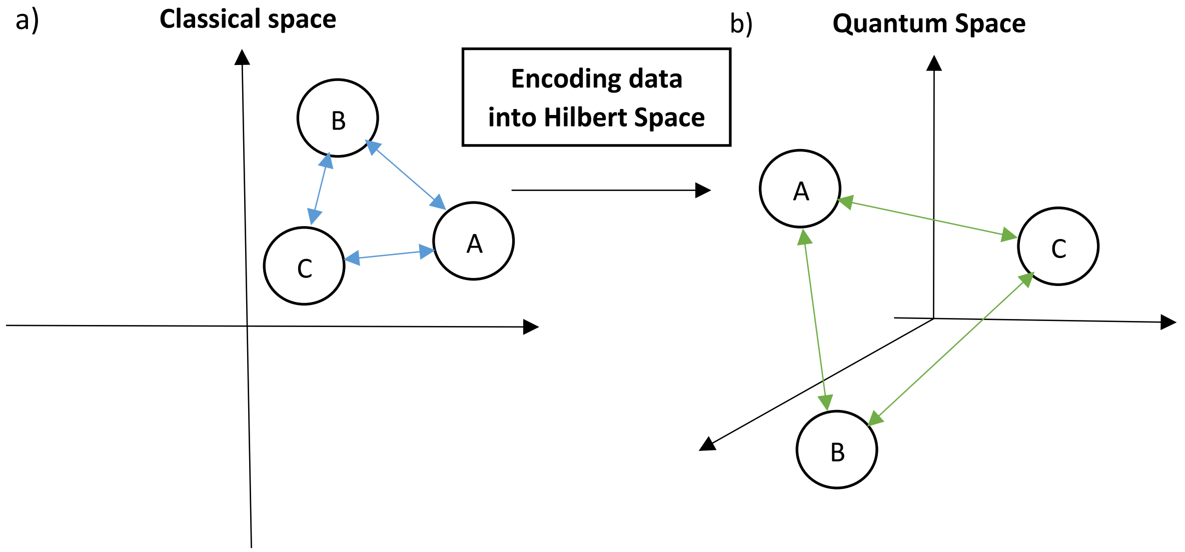

Figure S11. a) The position of three different data points A, B, and C in the classical space. **b)** After encoding classical data into quantum Hilbert space, the kernel entries corresponds to the data points in the quantum space decrease (distances increase). In this case the quantum advantage score g_{CQ} grows.

In the second case, if the classical kernel matrix corresponds to Fig. S12(a) is

$$K^C = \begin{pmatrix} 1 & 0.3 & 0.6 \\ 0.3 & 1 & 0.5 \\ 0.6 & 0.5 & 1 \end{pmatrix}$$

and the quantum kernel matrix corresponds to Fig. S12(b) is

$$K^Q = \begin{pmatrix} 1 & 0.9 & 0.8 \\ 0.9 & 1 & 0.7 \\ 0.8 & 0.7 & 1 \end{pmatrix}$$

then the $g_{CQ} = 1.2$.

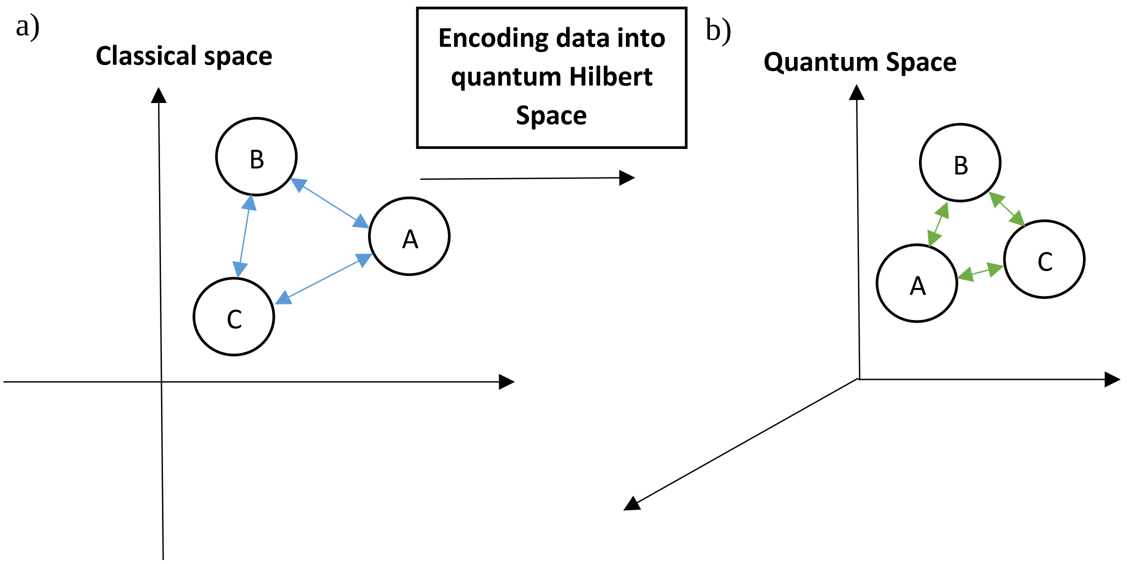

Figure S12. a) The position of three different data points A, B, and C in the classical space. **b)** After encoding classical data into quantum Hilbert space, the kernel entries correspond to the data points in the quantum space increase (distances decrease). In this case the quantum advantage score g_{CQ} decline.

Appendix F: Another method for estimation of $|\langle u|v\rangle|^2$ with $\log_2 N$ encoding in Pennylane simulator

To estimate the overlap $|\langle u|v\rangle|^2$, one can prepare the two states $|u\rangle$ and $|v\rangle$ on different sets of qubits with the $\log_2 N$ routines and then measure their overlap with the Swap Test (See equation (12) and Fig. 2 in manuscript). In general, if $N = 2^n$(N number of feature counts), the number of qubits is required to estimate $|\langle u|v\rangle|^2$ with the Swap Test are $1 + 2\log_2 N = 1 + 2n$. However, with the Pennylane simulator, one need only n number of qubits instead of $1 + 2n$If U is a data-encoding feature map, $K(\vec{x}_i, \vec{\tilde{x}})$ is defined as the inner product between two data encoding feature vectors $\rho(\vec{x}_i) = |u\rangle\langle u|$ and $\rho(\vec{\tilde{x}}) = |v\rangle\langle v|$ as following

$$K(\vec{x}_i, \vec{\tilde{x}}) = tr[\rho(\vec{x}_i)\rho(\vec{\tilde{x}})] = |\langle u|v\rangle|^2, \quad \text{(F1)}$$

where $|u\rangle = U(\vec{x}_i)|0\rangle^{\otimes n}$and $|v\rangle = U(\vec{\tilde{x}})|0\rangle^{\otimes n}$with $\vec{x}_i, \vec{\tilde{x}} \in R$,

and $\rho(\vec{x}_i) = |u\rangle\langle u|$, and $\rho(\vec{\tilde{x}}) = |v\rangle\langle v|$ are density matrices [6].Fig. S13 shows the execution of the quantum circuit for estimation of overlap $|\langle u|v\rangle|^2$ based on equation (F1) on IBMQ Melbourne machine for 16 feature counts.

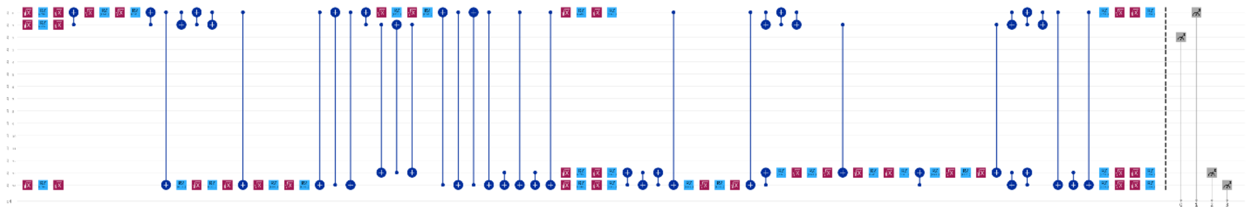

Figure S13. Quantum circuit to estimate the overlap $|\langle u|v\rangle|^2$. In our work, classical data are embedded in 4-qubit states. For mitigating the errors, instead of measuring the first qubit and compute the probability as the ratio of the number of times, IBMQ Melbourne machine measures all qubits.

Appendix G: Experimental vs. Simulator results in the Wisconsin Breast Dataset

Table S3 and S4 demonstrate the result of the inner product of the train and test state vectors in the Wisconsin Breast Cancer dataset from the simulator and the NISQ device. This dataset was chosen as it was providing the highest predictive cross-validation performance.

Simulator	Experiment
0.99731445	0.96112412
0.99975586	0.96103537
0.99804688	0.95926965
0.99072266	0.94346566
0.9987793	0.96225939
0.99902344	0.96659979
0.98510742	0.92951251
0.99584961	0.95624028
0.99780273	0.96399544
0.99780273	0.96595994
0.99804688	0.96006015
0.99389648	0.95516533
0.99755859	0.94856032
0.99975586	0.96524602
0.99951172	0.96814957
0.99975586	0.96419696
0.9987793	0.96087165
0.99975586	0.96539174
0.99780273	0.96595487
0.99902344	0.95822572
0.99975586	0.96650487
0.99951172	0.96373871
0.99951172	0.96193827
0.99853516	0.96626416
1.	0.96596886
0.99414062	0.94053438
0.99316406	0.94181723
1.	0.95599433
0.99731445	0.96254194
0.9987793	0.96467032
0.99975586	0.96855924
0.99902344	0.96524826
0.99902344	0.95855295

0.99536133	0.95312006
0.99951172	0.96590859
0.99926758	0.96571504
0.99707031	0.94822447
0.99951172	0.96891241
0.99609375	0.94659031
0.99926758	0.96632589
0.99731445	0.95831742
0.99804688	0.96289076

Table S3. The values of the inner product of the train and test state vectors (Wisconsin Breast Cancer dataset, 8 features, $\log_2 N$ encoding)

**Simulator**	**Experiment**
0.99707031	0.92630438
0.99267578	0.92135464
0.99389648	0.92650787
0.99243164	0.92750112
0.99462891	0.92948507
0.98608398	0.91441441
0.99731445	0.91670529
0.99072266	0.92623362
0.99316406	0.92795809
0.87133789	0.79162077
0.99365234	0.91339712
0.94775391	0.89294637
0.99829102	0.93250948
0.98828125	0.90181806
0.97509766	0.90796459
0.99487305	0.91907908
0.9921875	0.92437216
0.98413086	0.90482882
0.94091797	0.88528044
0.98730469	0.89584281
0.99487305	0.90275101
0.98144531	0.90580224
0.9934082	0.90776304
0.96777344	0.87167887
0.95507812	0.85660504

0.99414062	0.9236293
0.99121094	0.92107083
0.99829102	0.92467406
0.99609375	0.93314405
0.9855957	0.92024353
0.95336914	0.88968439
0.984375	0.90325062
0.99487305	0.91701626
0.96655273	0.88973655
0.98730469	0.90657753
0.98657227	0.91612964
0.99462891	0.91416633
0.99243164	0.92495982
0.98901367	0.91434396
0.9519043	0.86707464
0.98339844	0.91272665
0.98876953	0.91759276

Table S4. The values of the inner product of the train and test state vectors (Wisconsin Breast Cancer dataset, 16 features, $\log_2 N$ encoding)

In Table S5, we demonstrate the label of test dataset and the labels assigned to them by the k-nearest neighbour and the qDC algorithm for the case of two classes 0 and 1 in the 8-dimensional Wisconsin Breast cancer dataset. It can be seen that our qDC with NISQ device provides the correct labels for 38 out of 42 for 8-dimensional points, or an accuracy of 90.4% while with the same qDC algorithm with simulator 39 correct labels out of 42 for 8-dimesnional points are assigned with an accuracy of 92.8%.

1	1 1 1 1 1 1 0 0 0 0 0 0 0 1 1 1 0 1 0 1 1 1 1 0 1 0 0 0 1 1 1 1 0 0 1 0 0 1 0 0 0 0
2	1 1 1 0 0 1 0 0 0 0 1 0 0 1 1 1 0 1 0 1 1 1 1 0 1 0 0 0 1 1 1 1 0 0 1 0 0 1 0 0 0 0
3	1 1 1 0 0 1 0 0 0 1 1 0 0 1 1 1 0 1 0 1 1 1 1 0 1 0 0 0 1 1 1 1 0 0 1 0 0 1 0 0 0 0
4	1 1 1 0 0 1 0 0 0 0 0 0 0 1 1 1 0 1 0 1 1 1 1 0 1 0 0 1 1 1 1 1 0 0 1 0 0 1 0 0 0 0

Table S5. Comparison of labels assigned by quantum distance classifier (qDC) algorithm corresponds to Wisconsin Breast Cancer data with 8 features. The first line shows the labels of the test data. The second line shows the prediction results of qDC with simulator. The third line shows the labels assigned by the quantum distance classifier algorithm with NISQ device. The fourth line shows the result of classical k-nearest neighbour with $k = 5$. The labels (label) in green show where the classification with NISQ device differs from the classification with simulator. The labels

(label) in red show where the classification with NISQ device and the simulator differs from the label of the test data. The labels in blue show where the classical k- nearest neighbour differs from the label of the test data.

In Table S6 we demonstrate, the label of test dataset and the labels assigned to them by the k-nearest neighbour and the qDC algorithm for the case of two classes 0 and 1 in the 16-dimensional Wisconsin Breast cancer dataset. It can be seen that our qDC with NISQ device provides the correct labels for 38 out of 42 for 8-dimensional points, or an accuracy of 90.4% while with the same qDC algorithm with simulator 40 correct labels out of 42 for 16-dimesnional points are assigned with an accuracy of 95.2%.

Owing to noisy qubits and the errors of gate operations, the predictive performance from NISQ device reduces by 5% for Wisconsin Breast Cancer with 16 features, while the predictive performance reduces by almost 2% for Wisconsin Breast Cancer with 8 features.

1	1 1 0 1 0 0 1 1 0 1 1 0 1 1 1 1 1 1 1 1 0 0 0 0 0 1 1 1 1 0 0 0 0 1 0 0 0 0 1 0 0 0
2	1 1 0 1 0 0 1 0 0 1 0 0 1 1 1 1 1 1 1 1 0 0 0 0 0 1 1 1 1 0 0 0 0 1 0 0 0 0 1 0 0 0
3	1 1 0 1 0 1 1 0 0 1 0 0 1 1 1 1 1 1 1 1 0 0 0 0 1 1 1 1 1 0 0 0 0 1 0 0 0 0 1 0 0 0
4	1 1 0 1 0 0 1 0 0 1 0 0 1 1 1 1 1 1 1 1 0 0 0 0 0 1 1 1 1 0 0 0 0 1 0 0 0 0 1 0 0 0

Table S6. Comparison of labels assigned by quantum distance classifier (qDC) algorithm corresponds to Wisconsin Breast Cancer data with 16 features. The first line shows the labels of the test data. The second line shows the prediction results of qDC with simulator. The third line shows the labels assigned by the quantum distance classifier algorithm with NISQ device. The fourth line shows the result of classical k-nearest neighbour with $k = 5$. The labels (label) in green show where the quantum classification with NISQ device differs from the quantum classification with simulator. The labels (label) in red show where the quantum classification with NISQ device and the simulator differs from the label of the test data. The labels in blue show where the classical k- nearest neighbour differs from the label of the test data.

Appendix H: Implementation of the Hadamard Test on IBMQ Melbourne machine

The Hadamard Test approach is used to estimate the inner product of two state vectors. To map the quantum circuit of the Hadamard Test on the IBMQ Melbourne machine, first, we design the circuit in the Pennylane-Qiskit 0.13.0 environment. As can be seen from Fig. S14, there are Toffoli gates ($CCNOT$), and single control R_y gates which are needed to be decomposed before mapping the circuit of Fig. S14 on the IBMQ Melbourne machine architecture. To this end, we decompose each Toffoli gate in Fig. S14 into Hadamard (H), T, $T^{\dagger}$, and $CNOT$ gates and each single control R_y gates into single qubit rotation gates and two $CNOT$s (see Appendix B of the supplementary material). To design the Hadamard Test dataset with 16 features, $CNOT(q_0, q_4)$ cannot be implemented on the IBMQ Melbourne machine directly due to coupling constraints [7]. There are

two approaches to map $CNOT(q_0, q_4)$ on the IBMQ Melbourne. Let's first consider the physical qubits path Q_4 (root node), Q_{10}, Q_9, Q_8, and Q_7 (goal node) from Fig. 5 of manuscript as simulator qubits q_0, q_1, q_2, q_3, and q_4, respectively. The first option is to use a SWAP gate between Q_4 and Q_{10}, then, a $CNOT$ between Q_4 and Q_7. To complete the effect, a final SWAP between Q_{10} and Q_4 is applied [7,8]. Since each SWAP gate is implemented using three $CNOT$ gates, seven $CNOT$s are required to reproduce the effect of just one $CNOT(q_0, q_4)$. Another more efficient way is to only use sequences of $CNOT$ gates as following [8]

$$CNOT(Q_4, Q_7) = CNOT(Q_4, Q_{10})CNOT(Q_{10}, Q_7)CNOT(Q_4, Q_{10})CNOT(Q_{10}, Q_7), \quad \text{(H1)}$$

where $CNOT(q_0, q_4) = CNOT(Q_4, Q_7)$.

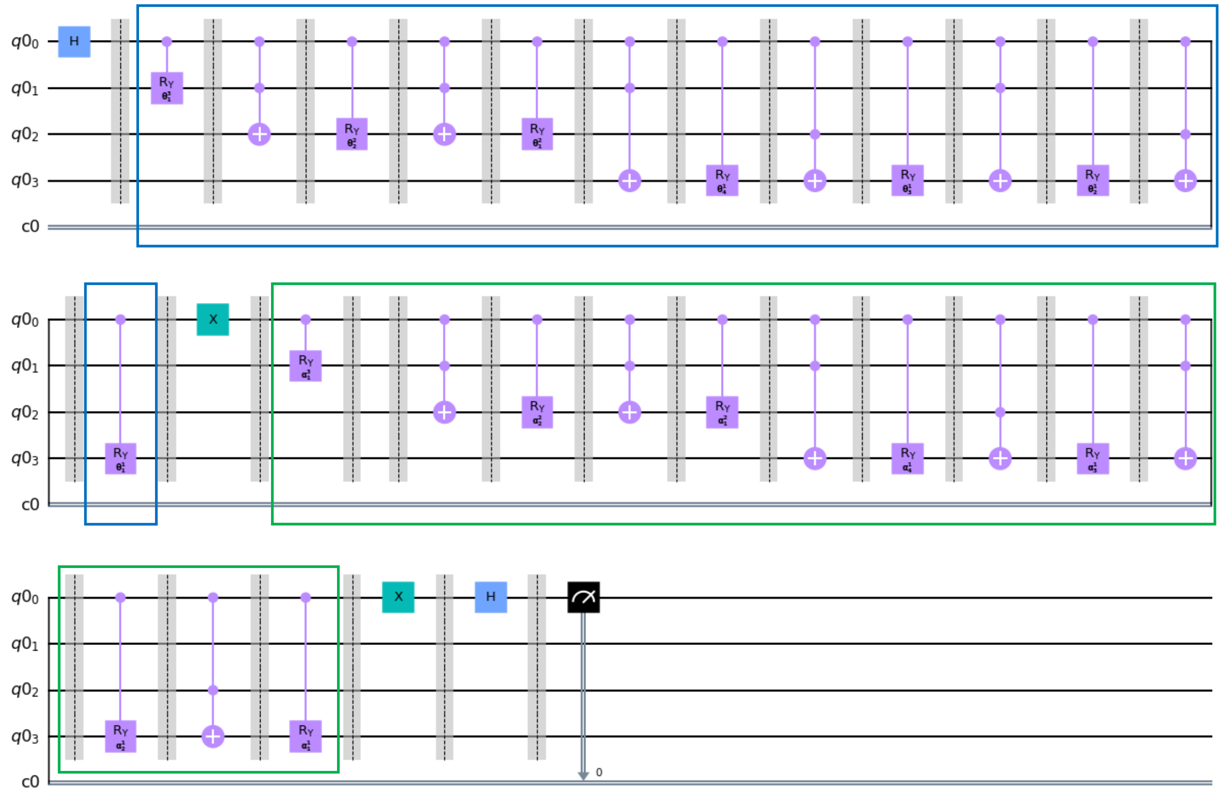

Figure S14. The Hadamard Test circuit to estimate the inner product of state vectors of the training and the test datasets with 8 features (3+1-qubit). The ancilla qubit ($q0_0$) is measured to estimate $\langle u|v\rangle$. The blue boxes are the part of quantum circuit that encodes the train data into the quantum state and entangles the ancilla qubit with the quantum state vector of the train data. The quantum circuit in the green boxes encode the test data and also entangle quantum state vector of the test data with the ancilla qubit.

References

1. Möttönen, M., Vartiainen, J. J., Bergholm, V. & Salomaa, M. M. Quantum Circuits for General Multiqubit Gates. *Phys. Rev. Lett.* **93**, 130502 (2004).

2. Schuld, M. & Petruccione, F. *Supervised Learning with Quantum Computers*. (Springer International Publishing, 2018). doi:10.1007/978-3-319-96424-9.

3. Niemann, P., Datta, R. & Wille, R. Logic Synthesis for Quantum State Generation. in *2016 IEEE 46th International Symposium on Multiple-Valued Logic (ISMVL)* 247–252 (IEEE, 2016). doi:10.1109/ISMVL.2016.30.

4. Schuld, M. & Killoran, N. Quantum Machine Learning in Feature Hilbert Spaces. *Phys. Rev. Lett.* **122**, 040504 (2019).

5. Havlíček, V. *et al.* Supervised learning with quantum-enhanced feature spaces. *Nature* **567**, 209–212 (2019).

6. Bergholm, V. *et al.* PennyLane: Automatic differentiation of hybrid quantum-classical computations. (2018).

7. Wille, R., Hillmich, S. & Burgholzer, L. Efficient and Correct Compilation of Quantum Circuits. in *2020 IEEE International Symposium on Circuits and Systems (ISCAS)* 1–5 (IEEE, 2020). doi:10.1109/ISCAS45731.2020.9180791.

8. Bataille, M. Quantum circuits of CNOT gates. (2020).

9. Huang, H.-Y. *et al.* Power of data in quantum machine learning. *Nat. Commun.* **12**, 2631 (2021).
